# Clinical and Genetic Study of Algerian Patients with Spinal Muscular Atrophy

**DOI:** 10.1155/2013/903875

**Published:** 2013-03-24

**Authors:** Y. Sifi, K. Sifi, A. Boulefkhad, N. Abadi, Z. Bouderda, R. Cheriet, M. Magen, J. P. Bonnefont, A. Munnich, C. Benlatreche, A. Hamri

**Affiliations:** ^1^Service of Neurology CHU of Constantine, Algeria; ^2^Laboratory of Biology and Molecular Genetics CHU and University of Constantine, Algeria; ^3^Laboratory of Biochemistry CHU of Constantine, Algeria; ^4^Service of Pediatrics CHU de Constantine, Algeria; ^5^Genetic Department of the Necker Hospital and Paris Descartes University, Paris, France

## Abstract

Spinal muscular atrophy (*SMA*) is the second most common lethal autosomal recessive disorder. It is divided into the acute Werdnig-Hoffmann disease (type I), the intermediate form (type II), the Kugelberg-Welander disease (type III), and the adult form (type IV). The gene involved in all four forms of *SMA*, the so-called survival motor neuron (*SMN*) gene, is duplicated, with a telomeric (tel *SMN* or *SMN*1) and a centromeric copy (cent *SMN* or *SMN*2). *SMN*1 is homozygously deleted in over 95% of *SMA* patients. Another candidate gene in *SMA* is the neuronal apoptosis inhibitory protein (*NAIP*) gene; it shows homozygous deletions in 45–67% of type I and 20–42% of type II/type III patients. Here we studied the *SMN* and *NAIP* genes in 92 Algerian *SMA* patients (20 type I, 16 type II, 53 type III, and 3 type IV) from 57 unrelated families, using a semiquantitative PCR approach. Homozygous deletions of *SMN*1 exons 7 and/or 8 were found in 75% of the families. Deletions of exon 4 and/or 5 of the *NAIP* gene were found in around 25%. Conversely, the quantitative analysis of *SMN*2 copies showed a significant correlation between *SMN*2 copy number and the type of *SMA*.

## 1. Introduction

Spinal muscular atrophies (*SMA*s) are a group of motor neuron disorders characterized by degeneration of spinal cord anterior horn cells, leading to muscular wasting and atrophy [1]. *SMA* is the most common autosomal recessive disorder after cystic fibrosis, with an estimated 1/10,000 incidence and a 1/60 carrier frequency [2]. Affected patients are classified into four groups according to age at onset and phenotype severity [3, 4]. Type I *SMA* or the Werdnig-Hoffmann disease (OMIM No. 253300) is the most severe form, with an onset within the first 6 months of age, severe generalized muscle weakness with hypotonia, and death before two years of age. In type II *SMA* (OMIM No. 253550), affected children sit unassisted, may be able to walk for a short distance, and usually survive over 10 years of age. Type III *SMA* or the Kugelberg-Welander disease (OMIM No. 253400) has its onset in the first to third decade. Though its course is highly variable, patients are constantly able to walk unassisted. Type IV *SMA* or adult-onset *SMA* (OMIM No. 271150) is quite rare.

The survival motor neuron (*SMN*) gene, implicated in the four forms of *SMA*, maps to chromosome 5q 11.2–13.3 [5–7] and is duplicated as telomeric and centromeric copies, so called *SMN*1 (OMIM No. 600354) and *SMN*2 (OMIM No. 601627), respectively [8, 9]. *SMN*1 and *SMN*2 comprising 8 exons are highly homologous, with only five base-pair differences within their 3′ ends [8, 10], and thus encode nearly identical proteins. Two of these base-pairs, located in exons 7 and 8, allow *SMN*1 to be distinguished from *SMN*2 at DNA and RNA levels and are currently used for detection of *SMN*1 deletions [11]. A vast majority (90–98%) of *SMA* patients have homozygous deletions of *SMN*1 exons 7 and 8 [8, 12, 13], the remaining ones carrying *SMN*1 intragenic mutations [8, 14, 15], with a frequency higher in type I than in types II and III.

Conversely, *SMN*2 homozygous inactivation is not directly responsible for *SMA* [8]. A number of studies have however shown that *SMN*2 acts as a modulator of *SMA* severity, with an inverse correlation between the *SMN*2 copy number and the disease severity [16, 17]. Failure of *SMN*2 to fully compensate homozygous loss of *SMN*1 is due to a sequence difference in exon 7 which causes alternative splicing of the *SMN*2 gene, and subsequently lower amount of full-length protein [8, 18, 19].

The neuronal apoptosis inhibitory protein gene (*NAIP*) [20], close to the *SMN* genes (15, 5 kb) at 5q11–q13, was initially considered as a candidate gene for *SMA* [12, 20, 21]. While subsequent studies have ruled out its direct responsibility, for this disease [17, 22], *NAIP* has however shown to be more frequently mutated in *SMA* affected patients than in general population, with homozygous deletions in 45–67% of typeI and 20–42% of typeII/typeIII *SMA* patients [12, 20, 23–25].

Analysis of deletions encompassing both *NAIP* and *SMN* genes in a large number of *SMA* patients suggests that loss of *NAIP* may be associated with a higher disease severity [10, 20]. Here we investigated the clinical and molecular characteristics of 92 Algerian *SMA* patients from 57 families to assess the prevalence of *SMN*1 deletions and the combined impact of *SMN*2 copy number and *NAIP* deletions on clinical severity.

## 2. Materials and Methods

### 2.1. Patients

92 patients from 57 Algerian families were diagnosed as having SMA on the basis of clinical findings and electromyoneurography. All patients fulfilled the diagnostic criteria for proximal SMA, as defined by the International SMA Collaboration [26] and by Zerres et al. [27] (Table 1). Inclusion and exclusion criteria were similar to those proposed by the International SMA Collaboration [26]. Patients with symmetrical, muscle weakness of trunk and limbs, proximal muscles weakness more than distal, lower limbs involvement more than upper limbs, and fasciculations of tongue and tremor of hands and in whom denervation was seen on EMG were included in our study. Patients who presented with CNS dysfunction, sensory loss, eye or facial muscle weakness, or involvement of other organs were excluded from this study. A complete clinical history was recorded with emphasis on age, sex, age at onset, course of the disease, perinatal history, parental consanguinity, and affected relatives. Clinical examination focused on neurological parameters, tone, power, reflexes, wasting and atrophy of muscles, and abnormal movements and sensations. Other investigations included serum creatine phosphokinase (CPK), electromyogram (EMG), and nerve conduction velocity. 

### 2.2. Methods

After informed consent, DNA was extracted from peripheral blood samples according to a standard technique [28].

#### 2.2.1. Molecular Analysis of *SMN* Genes

Search for *SMN*1 exons 7 and 8 deletions was performed by PCR and restriction enzyme digestion, as described in [29]. The *SMN*2 copy number was determined by Multiplex Ligation-dependent Probe Assay [30, 31].

#### 2.2.2. Molecular Analysis of the *NAIP* Gene

All individuals were also tested for exons 4 and 5 deletion of the *NAIP *gene. PCR conditions and primers used to amplify exons 4 and 5 were identical to those of Roy et al. [20].

## 3. Results

### 3.1. Clinical and Genealogical Findings

The rate of consanguineous marriage in this study was approximately 47%. Degrees of consanguinity are listed in Table 2.

Twenty-two of the 57 families (39%) were multiplex. The most common type in our cohort was type III, with fifty-three (53) affected cases from 31 families (60%), followed by type I with 20 cases from 14 families (25%). Frequency of the different types is summarized in Table 3.

In the *SMA* type I group, the age at onset varied from birth to 6 months, with an average of 5 ± 2, 5 months. All patients were mentally alert. The main symptom was severe hypotonia with poor limb mobility. Only two patients could hold up their heads, for a short period of time. Twelve patients (60%) died between 2 and 53 months of age, due to respiratory failure following respiratory tract infections. The remaining ones are still alive (the oldest patient is currently 72 months old). All patients with prolonged survival suffered from joint contractures caused by progressive muscular atrophy, spine deformation as scoliosis or kyphoscoliosis, and swallowing difficulties. The Bulbar symptoms were observed in 5 patients of them (Table 4). They were not able to cope with everyday routine and thus were totally family dependent. The DNA analysis showed that the *SMN*1 gene is interrupted in five out of six *SMA* type I patients with prolonged survival, and only one patient showed homozygous deletion of *NAIP* gene (Table 4). The *SMN*2 copy number was determined in 5 *SMA* type I patients carrying homozygous *SMN*1 deletions, and two of them (2/5) had two *SMN*2 copies, the remaining ones (3/6) carrying 3 *SMN*2 copies (Table 4). 

In the *SMA* type II group, age of onset ranged from 8 to 18 months (average 12, 7 ± 3, and 3 months). They were defined by ability to sit alone. Some children experienced early difficulty for sitting or rolling over (2 patients), while 3 patients were able to crawl and stand with support at a mean age of 22 months for a period of 4 months, and two patients were able to walk with support for a period of 6 months. None walked unaided. Scoliosis and contractures constantly developed in the patients who all became wheelchair dependent (6/16). In the type II group all patients are still alive, and 4/16 patients (25%) survive beyond age 15. In the *SMA* type III group, clinical onset ranged from the first year of life to the 3rd decade. Twenty-two patients (41, 5%) were confined to a wheelchair at ages ranging from 10 to 34 years, the remaining ones being still able to walk, with support. Hand tremor was found in 26 out of the 53 patients type III. Distal muscle weakness and/or amyotrophy was associated with the classical proximal defect, with frequent spine deformities and contractures in 39 patients. Life span was not significantly reduced. 

*SMA* type II and III patients coexisted within 2 families. In the three adult-onset *SMA* patients (type IV), age of onset ranged from 20 to 41 years (mean age of onset 30 ± 8 years). Adult *SMA* patients, except for patient 2, had very mild phenotypes, compared with the childhood onset. 

Blood CPK activity was normal in all *SMA* type I patients and was occasionally normal or slightly elevated in patients with type II (3 patients) or type III *SMA* (14 patients). In EMG examination the increased mean potentials, amplitude, duration, and area were stated. Maximal effort pattern in both proximal and distal muscles was reduced; spontaneous activity fibrillation and occasional fasciculations were present. Motor conduction velocity and sensory nerve conduction were normal.

### 3.2. Molecular Findings

Homozygous deletions of *SMN*1 exon 7, exon 8, or both were observed in 43/57 families (75%) with the following distribution: type I 11/14, type II 7/10, type III 24/31, and type IV 1/2. Among the 43 families with deletions, 36 had both exons 7 and 8 deleted, while four had deletions only of exon 7, and 3 patients carried only homozygous deletion restricted to *SMN*1 exon 8 (Table 5). Homozygous deletions of exons 4 and/or 5 of the *NAIP* gene were found in 4/14 type I, 2/10 type II, 9/31 type III, and 0/2 type IV families (Table 5). Homozygous *NAIP* deletions were constantly associated with homozygous *SMN*1 deletions. 

The *SMN*2 copy number was determined in patients carrying homozygous *SMN*1 deletions *n* = 62 (Table 6). 11/15 (73%) *SMA* type I patients had one or two *SMN*2 copies, the remaining ones carrying 3 *SMN*2 copies, 10/12 (83%) type II patients carried three or four *SMN*2 copies, the remaining ones having 2 *SMN*2 copies, and 32/33 (96%) type III patients carried three or four *SMN*2 copies. Finally, both adult onset patients carried at least 5 *SMN*2 copies (Table 6).

## 4. Discussion

We analyzed three genes implicated in *SMA*, namely, *SMN*1, *SMN*2, and *NAIP*, in a cohort of 92 *SMA* affected patients from 57 Algerian families, in an attempt at phenotype/genotype correlation. All patients fulfilled the diagnostic criteria for proximal *SMA*, as defined by the International *SMA* Collaboration [27] and were classified into four subgroups according to the criteria of Zerres et al. [26]. Twenty patients had type I, 16 patients type II, 53 type III, and 3 patients type IV *SMA*. Though clinical classification of *SMA* is helpful in providing medical care and prognostic assessment; it is however based on subjective and arbitrary parameters which may still be controversial and subject to errors. Zerres and Rudnik-Schöneborn [32], in a retrospective study of 445 *SMA* patients, found 106 cases (24%) that could not be classified and suggested subdividing type III *SMA* into two groups, resulting in a total of four *SMA* types. In the present study, clinical classification of patients into four groups, based on criteria of the International *SMA* collaboration [27] and of Zerres and Rudnik-Schöneborn [32], was possible for most patients. In these classifications, age at onset is classically considered to be predictive of the outcome. However, in 11 cases (12%) age at onset and/or death and motor milestones (ability to walk independently) did clearly overlap between two subsets. It is thus important to keep in mind the possibility of long-standing disease courses with an early onset of weakness compatible with a prolonged survival. For example, 6 patients with *SMA* type I survived over age two. In 5 patients, age at onset was before 18 months, which is characteristic of type II *SMA*, while walking capacities were compatible with *SMA* type III. Coexistence of various types of *SMA* (II and III) within a given family occurred in our series (2/57 families), as reported elsewhere [33, 34], in favor of a continuous spectrum in childhood *SMA*. Additionally we found a predominance of males to females (17 female/36 males) in type III *SMA*, as previously reported by Rudnik-Schöneborn et al. who suggested the presence of a female sparing factor [35]. Tazir and Geronimi reported the same fact in a much larger Algerian series in which chronic cases were predominant [36].

Consanguinity rate was 47% in our cohort, that is above the average reported in the Algerian general population (*≈*39%) [37]. Furthermore twenty-four families (42%) had a positive history of affected relatives. These data emphasize the importance of lowering the consanguinity rate and the value of genetic counseling and prenatal diagnosis for preventing *SMA* in our community.

From molecular point of view, the proportion of *SMN*1 homozygous deletions was 75% in our study. lower than those found in several other previously reported population studies [38–42] (Table 7).

Deletions involving both exons 7 and 8 were observed in 36 families (63%), being much more frequent than deletions restricted to exon 7 (4 families, 7%) or 8 (3 families, 5%), in agreement with previous investigations [8, 23, 42, 43].

Several authors reported a frequency of large deletions, encompassing both *SMN* and *NAIP* genes, higher in *SMA* type I than in the other types [12, 25, 43–46].

In our study the frequency of *NAIP* gene deletions was 28%, 20%, and 16% for type I, II, and III, respectively, and did apparently not influence the disease severity.

Moreover, a great proportion of severely affected patients harboured no *NAIP* deletion, and the same pattern of deletions (involving *SMN* and *NAIP* genes) was found among affected sibs with different phenotypes (*SMA* II and *SMA* III). This supports the hypothesis that other factors may regulate the severity of the clinical course in addition to the extent of the deletion [17, 47]. The *SMN*2 gene was consistently present as at least one copy in our series, thus contributing to some amount of *SMN* protein [46, 48]. It has previously been reported that most *SMN*2 transcripts lack exon 7 and are thus functionally defective, reinforcing the view that the disease is the result of an insufficient amount of intact *SMN* protein [49]. Interestingly, no patient has been diagnosed with a homozygous absence of both *SMN*1 and *SMN*2 gene so far, suggesting that a total absence of *SMN* would be lethal in utero.

The results of our quantitative analysis of *SMN*2 gene copies clearly show that the disease phenotype is influenced by the number of copies of the *SMN*2 gene, consistent with previous studies indicating that type II and III patients have on average a larger number of *SMN*2 copies than type I *SMA* patients [50–53]. In our series of 11 *SMA* type I patients who had a determination of the *SMN*2 copy number, the two patients with one *SMN*2 copy had a median survival of 5 months, whereas those with two and three *SMN*2 copies survived 8 and 23 months, respectively.

It is classically admitted that the *SMN*2 copy number is less than 3 in *SMA* type I and at least 3 in *SMA* type II, III, and IV [52–54]. Such a correlation between the number of *SMN*2 genes and the clinical phenotype is however not conclusive.

In conclusion, our results are in agreement with the general consensus that there is no correlation between the size of *SMN*1 deletions and the clinical severity of *SMA* and that there exists a close relationship between *SMN*2 copy number and *SMA* disease severity, suggesting that the determination of *SMN*2 copy number may be a good predictor of *SMA* disease type. We suggest that other still unknown factors may regulate the severity of the clinical course and influence phenotype expression. Our study additionally understanding the function of the *SMN* protein would probably be the key in unraveling the molecular basis of *SMA*.

## Figures and Tables

**Table 1:** Classification of autosomal recessive proximal spinal atrophy as defined by Zerres et al. [26].

Type	Definition
I	Never able to sit
II	Able to sit but not to walk
	Able to walk
III	(a) Onset before 3 years
	(b) Onset 3–30 years
IV	Onset after 30 years

**Table 2:** Consanguinity in Algerian *SMA* Families.

Degree of inbreeding	No. of families
2nd degree	7 (12,28%)
3rd degree	10 (17,54%)
4th degree	8 (14%)
Distant relatives	3 (5%)
Unrelated	29 (51%)

**Table 3:** Frequency of *SMA* types.

*SMA* type	No. of cases	No. of families
I	20	14
II	16	10
III	53	31
IV	3	2

Total	92	57

**Table 4:** Phenotypic and genotypic analysis of the 6 *SMA* type I index patients with prolonged survival.

Phenotypic and genotypic analysis	N° 1	N° 2	N° 3	N° 4	N° 5	N° 6
Sex	F	M	M	M	M	M
Consanguinity	−	−	−	+	−	+
Age at onset (months)	4	2	4	6	4	Birth
Age at diagnosis (year)	4	5	5	3	2	2
Age at last information (months)	50	60	72	39	48	41
Lifting of head	+	+	+	+	−	−
Hypotonia and muscle weakness	+	+	+	+	+	+
Deep tendon reflexes	−	−	−	−	−	−
Fasciculations	−	−	+	−	+	−
Bulbar symptoms	+	+	+	+	+	−
Breathing difficulties	+	−	−	−	−	−
Frequent pneumonia	+	+	+	+	+	+
Age at death (months)	54	−	−	−	−	−
*SMN*1 gene deletion	Yes	Yes	Yes	No	Yes	Yes
*NAIP* gene deletion	Yes	No	No	No	No	No
*SMN*2 copy number	2	2	3	−	3	3

**Table 5:** Distribution of homozygous deletion of *SMN*1 and *NAIP* genes according to the different types of *SMA*.

*N* of families	*SMN*1 gene deletion			*NAIP* gene deletion
	Exon 7	Exon 8	Exons 7 and 8	Exon 4/5
*SMA* I (*n* = 14)	2 (14%)	1 (7%)	8 (57%)	4 (28%)
*SMA* II (*n* = 10)	1 (10%)	0	6 (60%)	2 (20%)
*SMA* III (*n* = 31)	1 (3%)	2 (6%)	21 (67%)	9 (29%)
*SMA* IV (*n* = 2)	0	0	1 (50%)	0

**Table 6:** Analysis of the *SMN*2 copy number in the 62 patients with homozygous absence of the *SMN*1 gene.

*SMA* type	*N* patients	*SMN*2 copy number				
		1	2	3	4	5
I	15	2 (13%)	9 (60%)	4 (26%)	0	0
II	12	0	2 (16%)	7 (58%)	3 (25%)	0
III	33	0	0	5 (15%)	27 (81%)	1 (3%)
IV	2	0	0	0	0	2 (100%)

**Table 7:** Frequency of *SMN*1 homozygous deletion in *SMA* around the world.

References	Countries	*N* patients	*SMN *Del (%)
[38]	Korea 2001	37	32,43%
[39]	Vietnam 2003	17	41,17%
[40]	Johannesburg 2007	92	51%
[41]	Egypt 2001	33	55%
[42]	Russia 2001	57	65%
[33]	Brasilia 1999	87	69%
*Our study *	*Algeria 2009 *	*92 *	*75% *
[43]	India 2005	45	76%
[44]	Saudi Arabia 1997	16	82%
[55]	Morocco 2003	54	83,33%
[56]	Turkey 2000	60	85%
[57]	Germany 1995	195	90%
[58]	Spain 1995	54	91%
[59]	Holland 1995	103	93%
[60]	Japan 2002	32	94%
[61]	Tunis 2006	60	95%
[62]	Iran 2004	22	95,40%
[24]	UK 1995	140	97,80%
[63]	Kuwait 2001	46	97,82%
[8]	France 1995	229	98%
[64]	Taiwan 1995	42	100%

## Electronic Database Information

Accession numbers and the URL for data presented herein are as follows: Online Mendelian Inheritance in Man (OMIM), http://www.ncbi.nlm.nih.gov/Omim/ (for type I *SMA* [MIM 253300], type II *SMA* [MIM 253550], type III *SMA* [MIM 253400], *SMN1 *[MIM 600354], and *SMN2 *[MIM 601627]).
